# Modeling transmission of SARS-CoV-2 Omicron in China

**DOI:** 10.1038/s41591-022-01855-7

**Published:** 2022-05-10

**Authors:** Jun Cai, Xiaowei Deng, Juan Yang, Kaiyuan Sun, Hengcong Liu, Zhiyuan Chen, Cheng Peng, Xinhua Chen, Qianhui Wu, Junyi Zou, Ruijia Sun, Wen Zheng, Zeyao Zhao, Wanying Lu, Yuxia Liang, Xiaoyu Zhou, Marco Ajelli, Hongjie Yu

**Affiliations:** 1 School of Public Health, Fudan University, Key Laboratory of Public Health Safety, Ministry of Education, Shanghai, China; 2 Shanghai Institute of Infectious Disease and Biosecurity, Fudan University, Shanghai, China; 3 Division of International Epidemiology and Population Studies, Fogarty International Center, National Institutes of Health, Bethesda, MD USA; 4 Laboratory for Computational Epidemiology and Public Health, Department of Epidemiology and Biostatistics, Indiana University School of Public Health, Bloomington, IN USA

**Keywords:** Viral infection, Epidemiology

## Abstract

Having adopted a dynamic zero-COVID strategy to respond to SARS-CoV-2 variants with higher transmissibility since August 2021, China is now considering whether, and for how long, this policy can remain in place. The debate has thus shifted towards the identification of mitigation strategies for minimizing disruption to the healthcare system in the case of a nationwide epidemic. To this aim, we developed an age-structured stochastic compartmental susceptible-latent-infectious-removed-susceptible model of SARS-CoV-2 transmission calibrated on the initial growth phase for the 2022 Omicron outbreak in Shanghai, to project COVID-19 burden (that is, number of cases, patients requiring hospitalization and intensive care, and deaths) under hypothetical mitigation scenarios. The model also considers age-specific vaccine coverage data, vaccine efficacy against different clinical endpoints, waning of immunity, different antiviral therapies and nonpharmaceutical interventions. We find that the level of immunity induced by the March 2022 vaccination campaign would be insufficient to prevent an Omicron wave that would result in exceeding critical care capacity with a projected intensive care unit peak demand of 15.6 times the existing capacity and causing approximately 1.55 million deaths. However, we also estimate that protecting vulnerable individuals by ensuring accessibility to vaccines and antiviral therapies, and maintaining implementation of nonpharmaceutical interventions could be sufficient to prevent overwhelming the healthcare system, suggesting that these factors should be points of emphasis in future mitigation policies.

## Abstract (web summary)

Estimates from a new modeling study suggest that current levels of vaccine coverage in China are insufficient to prevent overwhelming the healthcare system, and that, if left untreated, a nationwide Omicron wave could result in up to 1.55 million deaths.

## Main

First discovered in Southern Africa in November 2021 (ref. ^1^), the Omicron variant of SARS-CoV-2 has spread swiftly across the world and replaced the Delta variant to become the dominant strain globally^2^. Omicron has demonstrated an increased transmissibility relative to Delta^1,3–5^ and immune escape capability^6,7^. Together with the progressive waning of the protection against the infection associated with previous infections and/or vaccination^8–12^, these characteristics have led to large Omicron epidemics in most countries^13^. Despite signs of a possibly lower clinical severity than Delta^14–18^, the sheer volume of Omicron infections has strained healthcare systems worldwide, including in the United States^19,20^ and the United Kingdom^21^. For instance, in the United Kingdom, the Omicron wave has led to higher infection rates than during the second wave in the winter of 2021, with substantial hospitalizations and deaths (over 1,000 deaths reported per week between 14 January and 4 February 2022)^21^.

After controlling the initial epidemic wave in Hubei in early 2020, China has deployed multilayer nonpharmaceutical intervention (NPI) protocols to contain sporadic COVID-19 outbreaks, largely introduced from international travelers. Maintaining a low infection rate in the general population throughout the pandemic has provided China time to mass immunize the population against SARS-CoV-2. As of 18 April 2022, 91.4% of the population aged ≥3 years has received the full primary schedule of the COVID-19 vaccination (either inactivated vaccines administered on a two-dose schedule, or recombinant subunit vaccines administered on a three-dose schedule or recombinant adenovirus type-5-vectored vaccines administered as a single dose); 53.7% of those vaccinated have received a booster shot^22^. However, vaccine-induced population immunity may be insufficient to prevent COVID-19 outbreaks. From 1 March to 22 April 2022, more than 500,000 local Omicron infections have been reported in almost all provinces across China, with most (about 93%) occurring in Shanghai^22^. To contain the highly infectious and immune evasive Omicron variant, additional NPI measures will be required to maintain the dynamic zero-COVID policy. This policy, adopted by China to respond to SARS-CoV-2 variants with higher transmissibility since August 2021, consists of a comprehensive set of measures to identify SARS-CoV-2 infections and stop any transmission chain, thus repeatedly zeroing local transmission^23^. Whether, and for how long, a zero-COVID policy can remain in place is questionable and, as recommended by the WHO^24^, every country should be prepared to chart its own path to transit from a pandemic to an endemic phase while accounting for local epidemiology, vaccination levels, population immunity and the strength of health systems. In this regard, as of May 2022, two approved antiviral treatments (BRII-196/BRII-198 combination and nirmatrelvir tablet/ritonavir tablet combination package) have been used in China, providing a new tool against COVID-19 (refs. ^25,26^).

**Extended Data Fig. 1:**
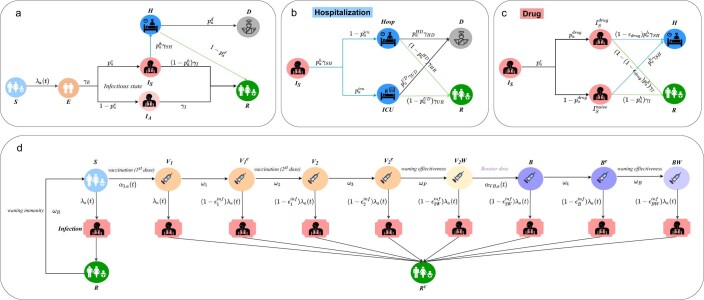
Model structure flow diagram with transition rates between states. **a**, Overview of extended SEIR transmission model. **b**, Diagram of hospitalization provided symptomatic infections. **c**, Diagram of antiviral therapies provided symptomatic infections. **d**, Diagram of vaccination and waning immunity. Note that the complex infection dynamic as shown in **a** has been encapsulated within a single pink box. All states and parameters are defined in Supplementary Tables 3 and 4.

**Extended Data Fig. 2:**
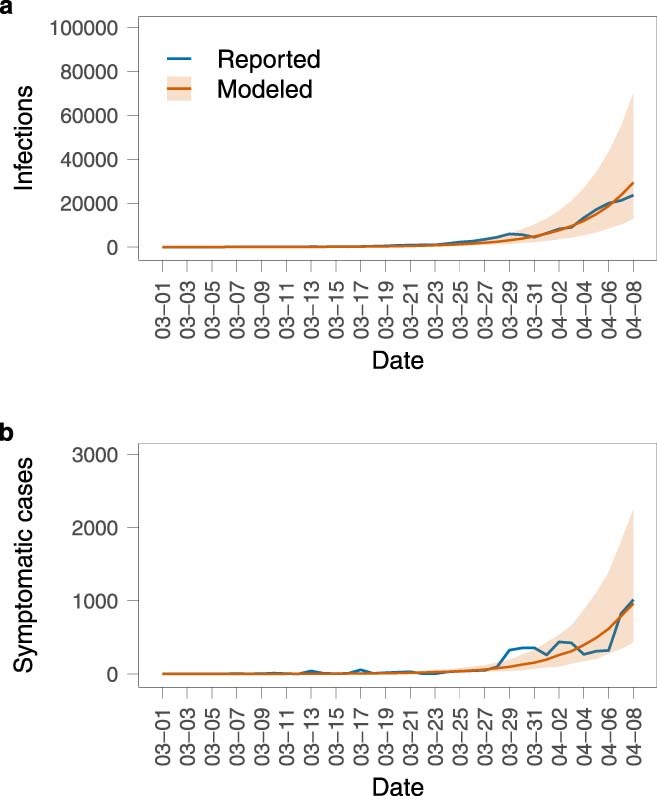
Comparisons between the observed epidemic curves in Shanghai and model output. **a**, Confirmed infections. **b**, Symptomatic infections. All data are presented as median with 2.5% and 97.5% quantiles of *n*=200 simulations.

Here, we explore the feasibility of a COVID-19 mitigation strategy to safeguard China’s shift from pandemic containment to mitigation, while minimizing the disease burden. Specifically, we leverage a mathematical model (Extended Data Fig. 1) to simulate a hypothetical Omicron wave in China based on data from the 2022 Omicron outbreak in Shanghai (Extended Data Fig. 2), project the demand for hospital beds and intensive care units (ICUs) and explore mitigation strategies combining vaccinations, antiviral therapies and NPIs to reduce COVID-19 burden while preventing the healthcare system being overwhelmed.

## Results

### Baseline scenario

The baseline scenario considers a homologous booster vaccination in the absence of strict NPIs and antiviral therapies. Specifically, the following conditions are simulated: (1) the introduction of 20 Omicron-infected individuals into the Chinese population on 1 March 2022; (2) the reproduction number (*R*) at the beginning of the simulation is set at 3.9 (when considering the partial protection of the population induced by vaccination, the reproduction number decreases to 3.4, in agreement with what we estimated for the early phase (from 1 March to 8 March 2022) of the epidemic in Shanghai (Extended Data Fig. 2), before strict control measures were implemented (Methods)); (3) booster doses of inactivated vaccines are rolled out at a speed of 5 million doses per day from 1 March 2022 (before that date the daily vaccination rates were informed by the cumulative number of doses administered in China); (4) 90% of individuals who have completed the primary vaccination schedule by at least 6 months receive a booster shot; (5) vaccine efficacy (VE) is set according to the values reported in Supplementary Table 1, considering a low immune escape scenario with same VEs against hospitalization and deaths between homologous booster and heterologous booster vaccination as observed in Hong Kong^27^ and (6) antiviral therapies are not distributed.

**Fig. 1:**
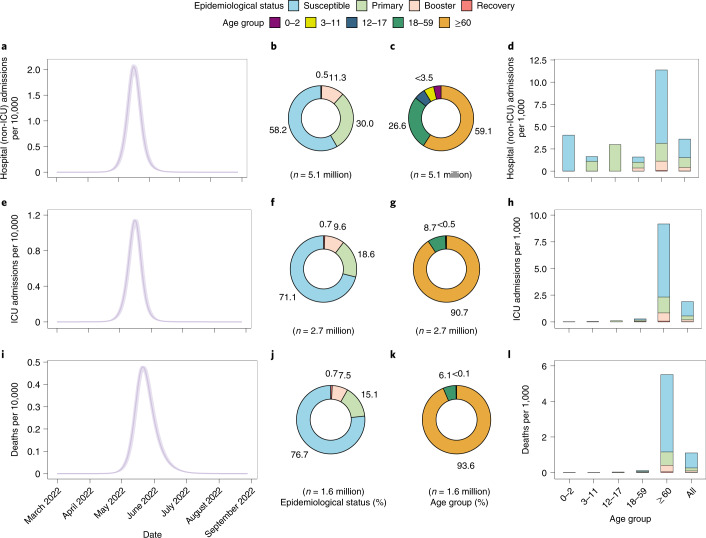
Projected SARS-CoV-2 Omicron burden in China for baseline scenario from March 2022 to September 2022. **a**, Daily hospital (non-ICU) admissions per 10,000 individuals. **b**, Epidemiological status of hospitalized (non-ICU) patients. **c**, Age distribution of hospitalized (non-ICU) patients. **d**, Distribution of hospitalized (non-ICU) patients per 10,000 by age group and epidemiological status. **e**, Daily ICU admissions per 10,000 individuals. **f**, Epidemiological status of ICU patients. **g**, Age distribution of ICU patients. **h**, Distribution of ICU patients per 10,000 by age group and epidemiological status. **i**, Daily deaths per 10,000 individuals. **j**, Epidemiological status of deaths. **k**, Age distribution of deaths. **l**, Distribution of deaths per 10,000 by age group and epidemiological status. In panels **b**, **f** and **j**, susceptible refers to individuals who do not receive COVID-19 vaccines; primary refers to those individuals who have received at least one dose of COVID-19 vaccine, considering a primary vaccination which entails a two-dose schedule; booster refers to those individuals who have received a third dose; recovery refers to individuals who have recovered from SARS-CoV-2 Omicron infection. Data are presented as median with 2.5% and 97.5% quantiles of *n* = 200 simulations.

**Fig. 2:**
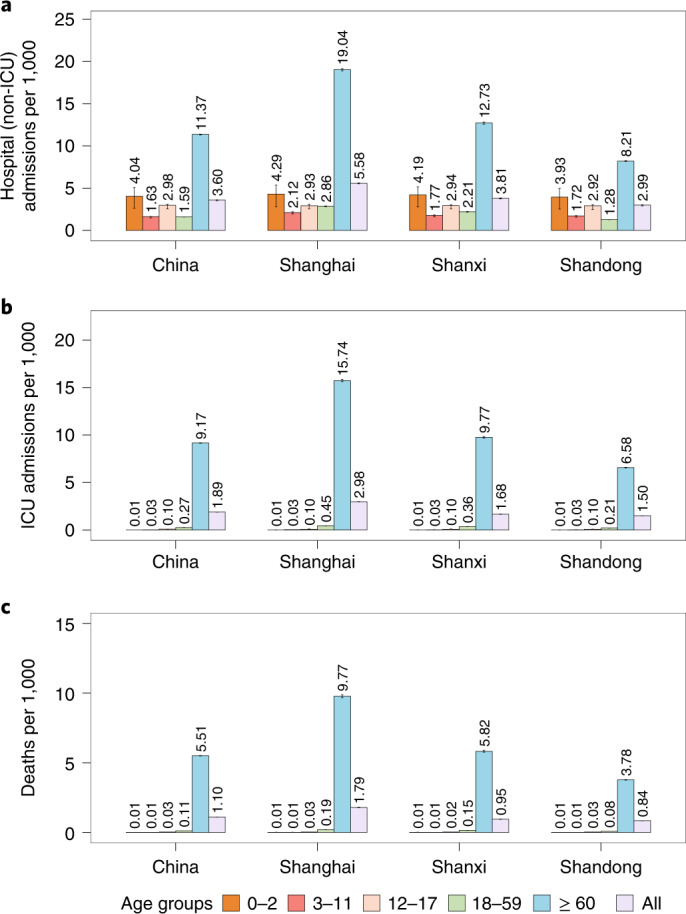
Age-specific and overall incidence rates of different clinical outcomes across four settings (China, Shanghai, Shanxi and Shandong) under the baseline scenario from March 2022 to September 2022. **a**, Cumulative hospital (non-ICU) admissions per 1,000 individuals. **b**, Cumulative ICU admissions per 1,000 individuals. **c**, Cumulative deaths per 1,000 individuals. China represents the ‘national average’. Number denotes median, and error bars denote 2.5% and 97.5% quantiles of *n* = 200 simulations.

Our simulated baseline scenario suggests that, in the absence of strict NPIs, the introduction of the Omicron variant in China in March 2022 could have the potential to generate a tsunami of COVID-19 cases. Over a 6-month simulation period, such an epidemic is projected to cause 112.2 million symptomatic cases (79.58 per 1,000 individuals), 5.1 million hospital (non-ICU) admissions (3.60 per 1,000 individuals), 2.7 million ICU admissions (1.89 per 1,000 individuals) and 1.6 million deaths (1.10 per 1,000 individuals), with a main wave occurring between May and July 2022 (Figs. 1 and 2).

According to our model simulations, 41.3% of non-ICU hospitalizations and 28.2% of ICU admissions would occur among vaccinated individuals. Most non-ICU hospitalizations are estimated to occur in the adult population (26.6% among individuals aged 18–59 years and 59.1% among individuals aged ≥60 years), while over 90% of ICU admissions would occur among individuals aged ≥60 years (Fig. 1). Most deaths (76.7%) are estimated to occur among nonvaccinated individuals, despite representing only 12.1% of the population (Fig. 1). Unvaccinated individuals aged ≥60 years are projected to account for 74.7% of the total number of deaths due to the gap in vaccination coverage in this portion of the population; approximately 52 million people aged ≥60 years are not fully vaccinated as of 17 March 2022 (ref. ^28^).

In addition to presenting analyses for a national average, we analyze three highly diverse areas of China: Shanghai, Shandong and Shanxi. For each of these areas, we consider a specific vaccination coverage, age structure of the population, contact patterns of the population (Supplementary Fig. 1) and number of available hospital beds and ICUs (Supplementary Table 2). The results show a considerable heterogeneity across the different areas. For example, the number of deaths per 1,000 inhabitants in the baseline scenario is projected to be 1.79 in Shanghai (where, as of 15 April 2022, 62% of individuals aged ≥60 years were fully vaccinated and 38% had received a booster dose^29^) compared with 0.84 in Shandong (the province with the highest vaccination coverage in individuals aged ≥60 years among the four settings; as of 21 March 2022, 89.16% of this population were fully vaccinated and 72.45% had received a booster dose^30^) (Fig. 2).

**Fig. 3:**
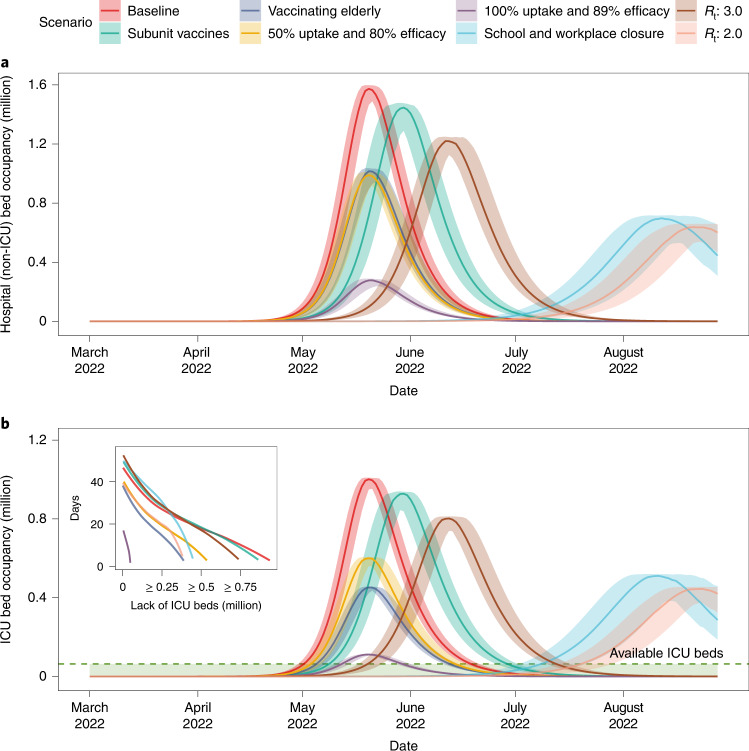
Projected demand and shortage of hospital beds and ICUs when adopting individual mitigation strategies in China under optimistic VE scenario from March 2022 to September 2022. **a**, Daily demand of hospital (non-ICU) beds. **b**, Daily demand of ICU beds. In **b**, the green dashed line indicates the number of ICU beds available in China, and the inset plot shows days of shortage of ICU beds as a function of the number of insufficient ICU beds compared with the capacity of ICU beds. The curves in the inset are smoothed by B-spline with 8 d.f. The scenarios included in legend are as follows: subunit vaccines refer to using a third dose of subunit vaccines as booster after two doses of inactivated vaccines as priming. Vaccinating elderly refers to vaccinating approximately 52 million people aged ≥60 years who have not yet been vaccinated as of 17 March 2022. 50% uptake and 80% efficacy corresponds to a scenario where 50% of symptomatic cases receive an antiviral therapy with an efficacy of 80% in preventing hospitalization and death. 100% uptake and 89% efficacy corresponds to a scenario where all symptomatic cases receive an antiviral therapy with an efficacy of 89% in preventing hospitalization and death. School and workplace closure corresponds to a scenario where, on the top of baseline strategy, all schools and workplaces remain closed for the duration of the epidemic. *R*_*t*_: 3.0 and 2.0 correspond to scenarios assuming different levels of NPIs leading to reduced values of the reproduction number. Note that no strict NPI is implemented in the baseline scenario. Data are presented as median with 2.5% and 97.5% quantiles of *n* = 200 simulations.

**Extended Data Fig. 3:**
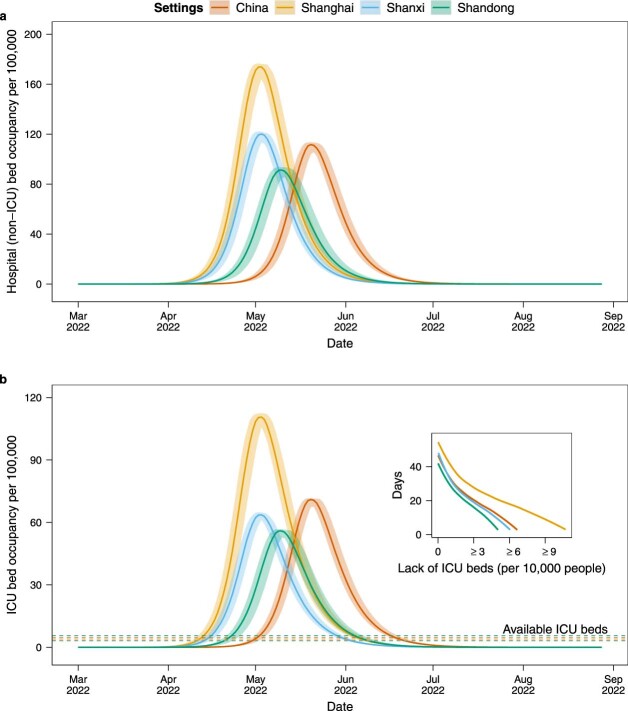
Projected demand and shortage of hospital beds and ICUs for baseline scenario across four settings from March 2022 to September 2022. **a**, Daily demand of hospital (non-ICU) beds per 100,000. **b**, Daily demand of ICU beds per 100,000. In **b**, the dashed lines indicate the existing number of ICU beds per 100,000 in four settings, and the inset plot shows days of shortage of ICU beds as a function of the number of insufficient ICU beds compared with the capacity of ICU beds in each setting. The curves in the inset are smoothed by B-spline with 8 d.f. China represents the ‘national average’. All data are presented as median with 2.5% and 97.5% quantiles of *n*=200 simulations.

To evaluate the impact of an uncontrolled Omicron epidemic on the national healthcare system, we considered that all COVID-19 hospitalizations require hospital beds for respiratory illness and that critically ill cases require ICU beds, and computed the corresponding demands. At the national scale, it is estimated that 1.57 million hospital beds for respiratory illness would be required at the epidemic peak, which is fewer than the number of existing hospital beds for respiratory illness (3.1 million) in China^31^. However, the peak demand of ICU beds (1.00 million) corresponds to 15.6 times the number of existing ICU beds in China (that is, 64,000)^31^. The period of ICU bed shortage is estimated to last for approximately 44 days (Fig. 3). In the regional analyses, substantial shortages of ICU beds were also predicted to occur in Shanghai, Shandong and Shanxi province (Extended Data Fig. 3).

**Extended Data Fig. 4:**
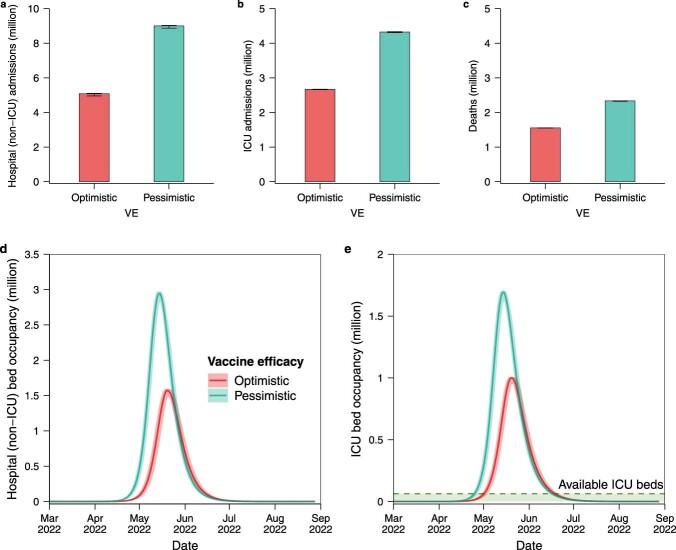
Projected COVID-19 burden and demands in hospital and ICU beds in China under pessimistic VE scenario compared to the baseline scenario (optimistic VE) from March 2022 to September 2022. **a**, Cumulative hospital (non-ICU) admissions. **b**, Cumulative ICU admissions. **c**, Cumulative deaths. **d**, Daily demand in hospital (non-ICU) beds. **e**, Daily demand in ICU beds. In **e**, the green dashed line indicates the number of ICU beds available in China. All data are presented as median with 2.5% and 97.5% quantiles of *n*=200 simulations.

When considering a more conservative scenario on the immune escape of the Omicron variant (referred as to high immune escape scenario), with (1) lower VEs against all clinical endpoints as compared with low immune escape scenario, and (2) lower VEs against hospitalization and deaths for homologous booster as compared with heterologous booster vaccination (as observed in Brazil^32^, Supplementary Table 1), the projected number of hospitalizations, ICU admissions and deaths at the national level would increase by 77.3%, 62.1% and 50.2%, respectively (Extended Data Fig. 4).

### Impact of individual mitigation strategies

**Fig. 4:**
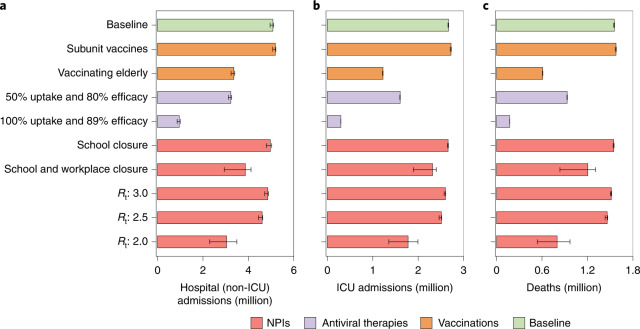
Projected impact of adopting individual mitigation strategies on COVID-19 burden in China under optimistic VE scenario from March 2022 to September 2022. **a**, Cumulative number of hospital (non-ICU) admissions. **b**, Cumulative number of ICU admissions. **c**, Cumulative number of deaths. The scenarios indicated on the *y* axis are as follows: subunit vaccines refer to using a third dose of subunit vaccines as booster after two doses of inactivated vaccines as priming. Vaccinating elderly refers to vaccinating approximately 52 million people aged ≥60 years have not been vaccinated yet as of 17 March 2022. 50% uptake and 80% efficacy corresponds to a scenario where 50% of symptomatic cases receive an antiviral therapy with an efficacy of 80% in preventing hospitalization and death. 100% uptake and 89% efficacy corresponds to a scenario where all symptomatic cases receive an antiviral therapy with an efficacy of 89% in preventing hospitalization and death. School closure corresponds to a scenario where, on the top of baseline strategy, all schools remain closed for the duration of the epidemic. Similarly, school and workplace closure corresponds to a scenario, where on the top of baseline strategy, all schools and workplaces remain closed for the duration of the epidemic. *R*_*t*_: 3.0, 2.5 and 2.0 correspond to scenarios assuming different levels of NPIs leading to reduced values of the reproduction number. Note that no strict NPI is implemented in the baseline scenario. Data are presented as median with 2.5% and 97.5% quantiles of *n* = 200 simulations.

**Extended Data Fig. 5:**
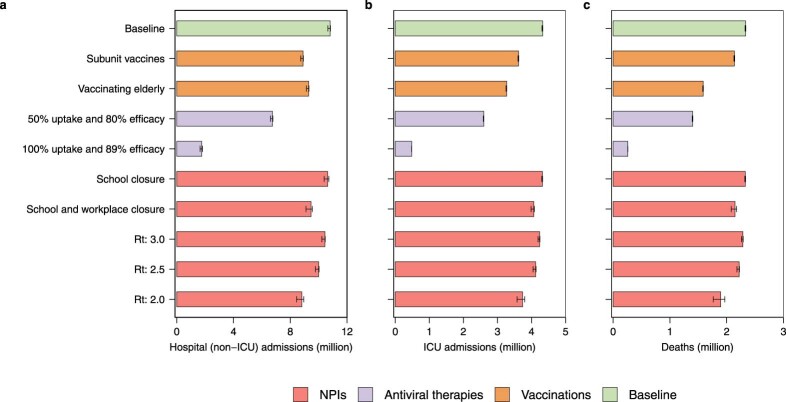
Projected impact of adopting individual mitigation strategies on COVID-19 burden in China under pessimistic VE scenario from March 2022 to September 2022. **a**, Cumulative number of hospital (non-ICU) admissions. **b**, Cumulative number of ICU admissions. **c**, Cumulative number of deaths. The scenarios indicated on *y* axis are as follows: subunit vaccines refer to using a third dose of subunit vaccines as booster after two doses of inactivated vaccines as priming. Vaccinating elderly refers to vaccinating approximately 52 million people aged ≥60 years who have not been vaccinated yet as of March 17, 2022. 50% uptake and 80% efficacy corresponds to a scenario where 50% of symptomatic cases receive an antiviral therapy with an efficacy of 80% in preventing hospitalization and death. 100% uptake and 89% efficacy corresponds to a scenario where all symptomatic cases receive an antiviral therapy with an efficacy of 89% in preventing hospitalization and death. School closure corresponds to a scenario where, on the top of baseline strategy, all schools remain closed for the duration of the epidemic. Similarly, school and workplace closure corresponds to a scenario, where on the top of baseline strategy, all schools and workplaces remain closed for the duration of the epidemic. *R*_*t*_: 3.0, 2.5, and 2.0 correspond to scenarios assuming different levels of NPIs leading to reduced values of the reproduction number. Note that no strict NPIs is implemented in the baseline scenario. All data are presented as median with 2.5% and 97.5% quantiles of *n*=200 simulations.

We investigated separately the impact of three categories of strategies to mitigate COVID-19 burden: (1) vaccination, including heterologous booster doses and promoting vaccination coverage among unvaccinated individuals aged ≥60 years, (2) antiviral therapies and (3) NPIs. Regarding booster vaccination, if we consider the administration of a heterologous booster based on a subunit vaccine (subunit vaccines scenario) in the low immune escape scenario, little difference would be observed in terms of COVID-19 burden (Fig. 4); on the other hand, in the high immune escape scenario, a larger decrease of COVID-19 burden (8.4% in the number of deaths and 17.7% in the number of hospital admissions) could be achieved by administrating a heterologous booster based on a subunit vaccine (Extended Data Fig. 5). Filling the gap in the vaccination coverage among the elderly (that is, vaccinating all eligible individuals aged 60 years or more), including both primary and booster vaccination as in the baseline scenario (vaccinating elderly scenario) would lead to a 33.8%, 54.1% and 60.8% decrease in hospital admissions, ICU admissions and deaths, respectively (Fig. 4).

In the absence of NPIs, assuming that 50% of symptomatic cases could be treated with the approved Chinese COVID-19 BRII-196/BRII-198 combination therapy, which has been reported to be 80% effective in preventing hospitalization and death^33^, a 36.5%, 39.9% and 40.0% decrease in hospital admissions, ICU admissions and deaths is estimated (50% uptake and 80% efficacy scenario). In the best-case scenario in which all symptomatic cases are treated with the highly efficacious oral COVID-19 drug nirmatrelvir tablet/ritonavir tablet combination (which is 89% effective in preventing hospitalization and death^34^ and has already been used in China^26^), the number of hospital admissions, ICU admissions and deaths could be reduced substantially by 81.2%, 88.8% and 88.9% (100% uptake and 89% efficacy scenario) (Fig. 4).

We then modeled the impact of introducing different levels of NPIs (in the presence of vaccination, but absence of antiviral therapies). First, we tested the implementation of a national-level school closure strategy (school closure scenario); although the number of infections decrease by 3.5%, COVID-19 burden does not, due to a shift in the age distribution of infections towards older ages. Additionally, closing all workplaces (school and workplace closure scenario) would lead to a decrease of 23.8%, 13.1% and 22.4% for the number of hospitalizations, ICU admissions and deaths, respectively. Second, we considered a scenario where NPIs equally reduce the risk of infection across all age groups, and we simulated different intensity of NPIs leading to *R*_*t* _≤ 3 (similar to values observed in England^35^ and India^36^ during the Omicron wave in winter 2021–2022). In this scenario, only the adoption of NPIs capable of reducing *R*_*t*_ to values ≤2 would lead to a substantial decrease in health outcomes (namely, a decrease of 40.1%, 33.4% and 48.6% of the number of hospitalizations, ICU admissions and deaths, respectively) (Fig. 4).

In summary, none of the scenarios analyzed is estimated to have the potential to reduce the number of COVID-19 deaths to a level closer to the annual influenza-related excess deaths in China (88,000)^37^ (Fig. 4). In all scenarios, the peak demand for ICUs is projected to be 1.7–14.8 times the maximum capacity, with a total of 19–48 days of bed shortages (Fig. 3b). We emphasize that closing all schools and workplaces as well as implementing stringent NPIs to reduce *R*_*t*_ to 2 would result in highly delayed epidemics that extend beyond our projection window (6 months); as such, their final impact is not evaluated in this analysis (Fig. 3b).

### Impact of combined mitigation strategies

**Fig. 5:**
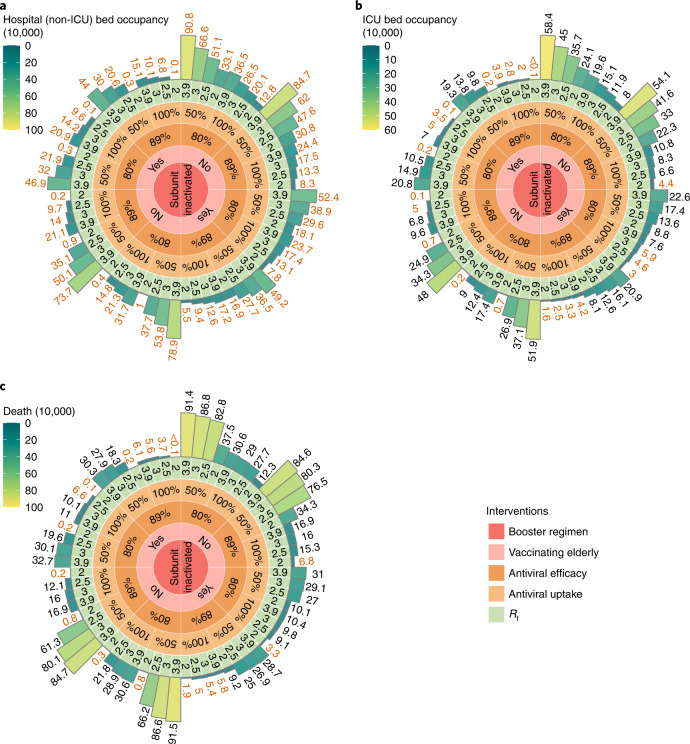
Projected healthcare demand and number of deaths for combined mitigation strategies under optimistic VE scenario in China from March 2022 to September 2022. **a**, Peak hospital (non-ICU) bed occupancy, with red numbers indicating where peak hospital bed demand is lower than the bed capacity for respiratory illness in China. **b**, Peak ICU bed occupancy, with red numbers indicating where peak ICU bed demand is below the existing ICU capacity in China. **c**, Cumulative death tolls, with red numbers indicating where the number of deaths is below the annual influenza-related excess death toll in China (that is, 88,000 deaths^37^). The circular-Manhattan plot from the innermost concentric circle to the outermost concentric circle indicates the combinations of adopting different intervention measures: homologous (inactivated) or heterologous (subunit) booster regimen; whether the approximately 52 million people aged ≥60 years who have not been vaccinated yet as of 17 March 2022 are vaccinated or not; receiving antiviral therapies with an efficacy of 80% or 89% in preventing hospitalization and death; 50% or 100% symptomatic cases receiving an antiviral therapy; *R*_*t*_ representing varying intensity of NPIs. *R*_*t*_ = 3.9 corresponds to the scenario in the absence of strict NPIs. Data are presented as median of *n* = 200 simulations.

None of the investigated individual mitigation strategies alone is capable of reducing the death toll to the level of an influenza season or to prevent exceeding critical care capacity (Figs. 3 and 4). Here, we assessed the effects of synergetic strategies leveraging heterologous booster vaccination, increasing vaccination coverage among the unvaccinated individuals aged 60 years or more, distributions of antiviral therapies and adoption of NPIs at the same time (Fig. 5).

None of the simulated interventions is projected to exceed the national capacity of hospital bed capacity for respiratory illness. Instead, a synergetic effort of combining different strategies would be needed to prevent exceeding ICU capacity and limiting the number of deaths to a value comparable to that of seasonal influenza. According to our analysis, key aspects of this synergetic effort are the increase of vaccine uptake in the elderly and the widespread use of antiviral therapies (Fig. 5). If these two conditions are not met, relying on NPIs capable of reducing *R* to ≤2 is needed to prevent overwhelming the healthcare system.

## Discussion

Using a stochastic dynamic model of SARS-CoV-2 transmission, our study projects the COVID-19 burden caused by the importation of Omicron infections in mainland China, should the dynamic zero-COVID policy be lifted. In the context of the vaccination strategy adopted until March 2022, we estimated that the introduction of the Omicron variant would cause substantial surges in hospitalizations, ICU admissions and deaths, and would overwhelm the healthcare system with an estimated burden of 15.6 times the available ICU capacity.

Should an Omicron variant epidemic be allowed to spread uncontrolled in mainland China, we project 1.10 deaths per 1,000 inhabitants over a 6-month period. By comparison, 187,372 deaths have been reported in the United States^38^ (that is, 0.57 deaths per 1,000 inhabitants) over the period from 15 December 2021 to 15 April 2022, roughly corresponding to the Omicron wave. We estimate that around 77% of the death toll in China would occur in unvaccinated individuals, with most deaths occurring among unvaccinated individuals aged 60 years or more (52 million people). A similar trend has been observed in the Omicron-driven fifth COVID-19 wave in Hong Kong Special Administrative Region (SAR) of China, which began in early 2022 (ref. ^39^). Our findings highlight the key role of increasing vaccine uptake rate among the elderly to limit COVID-19 burden and to prevent overwhelming the healthcare system. A second key factor to reach these goals is represented by the widespread and timely distribution of a highly efficacious antiviral therapy. When both vaccine uptake in the elderly is substantially increased (97%) and 50% or more of symptomatic infections are treated with antiviral therapies, the peak occupancy of ICUs may not exceed the national capacity and the death toll may be comparable to that of seasonal influenza. In the absence of these two conditions, the most optimistic strategy to prevent overwhelming of the healthcare system seems to be reliance on strict NPIs.

China is a highly diverse country with urban megalopolises on the eastern seaboard and rural areas in the northwest. Such diversity is also reflected by heterogeneous vaccination coverage, demographic structure of the population, mixing patterns and capacity of the healthcare system. When accounting for these heterogeneities, our simulations show considerable differences in the projected COVID-19 burden for different areas of China. According to our projections, the population of Shanghai would experience a COVID-19 burden higher than that of other areas such as Shandong and Shanxi. This increased burden would be led by a much larger incidence of severe infections in the population aged 60 years or older, which is associated with a lower vaccination coverage in this segment of the population. This result confirms the importance of filling the vaccination gap among the elderly and the need to tailor interventions to the specific immunological landscape of the population.

Our study has several limitations. First, we assumed that the mortality rate remains constant over the projection period; however, studies have suggested that the mortality rate may increase during periods of high strain on hospital services^40,41^. Second, although we conducted a comprehensive literature search, the epidemiological characteristics of Omicron, clinical severity, VEs of primary and booster vaccination and its persistence against different clinical endpoints, as well as the effectiveness of antiviral therapies, are not fully understood. For this reason, we have conducted extensive sensitivity analyses to explore the impact of the uncertainty of model parameters. Third, data on antiviral therapy availability by region are unknown and thus not included in our analysis. Possible regional differences in stockpiles of antiviral therapies could widen the already large differences in COVID-19 burden that we have estimated among the study locations.

In conclusion, should the Omicron outbreak continue unabated, despite a primary vaccination coverage of ≥90% and homologous booster vaccination coverage of ≥40% as of March 2022, we project that the Chinese healthcare system will be overwhelmed with a considerable shortage of ICUs. The contemporary increasing of vaccine uptake in the elderly and widespread distribution of antiviral therapies or the implementation of strict NPIs would be needed to prevent overwhelming of the healthcare system and to reduce the death toll of an epidemic wave to a level comparable with that of an influenza season. Protecting vulnerable individuals by ensuring access to vaccination and antiviral therapies, as well as maintaining implementation of NPIs (for example, mask-wearing, enhanced testing, social distancing and reducing mass gatherings), should be emphasized, together with tailoring region-specific interventions. In the long term, improving ventilation, strengthening critical care capacity and the development of new highly efficacious vaccines with long-term immune persistence would be key priorities.

## Methods

This modeling study relies on publicly available aggregated data only. As such, institutional review and informed consent are waived by the Institutional Review Board of the School of Public Health, Fudan University (Shanghai, China).

### Model SARS-CoV-2 transmission and vaccination

We developed an age-structured stochastic compartmental susceptible-latent-infectious-removed-susceptible model (Extended Data Fig. 1) to simulate the transmission of the SARS-CoV-2 Omicron variant in China. The model considers 14 age groups (0–2, 3–11, 12–17, 18–24, 25–29, 30–34, 35–39, 40–44, 45–49, 50–54, 55–59, 60–64, 65–69 and ≥70 years) and age-mixing patterns for China before the COVID-19 pandemic^42^. The model accounts for primary and booster vaccination, disease progression, antiviral therapies and waning immunity. All compartments and parameters are defined in Supplementary Tables 3 and 4. Transitions between compartments are simulated through a stochastic chain binomial process^43^. For instance, susceptible individuals move to the latent compartment at the rate $\Delta _\mathrm{a}(t)\sim \mathrm{Binomial}\left( {S_\mathrm{a}(t),1 - {\mathrm{e}}^{ - \lambda _\mathrm{a}\left( t \right)}} \right)$, where *λ*_a_(*t*) is the force of infection for age group *a* at time *t*.

Baseline simulations were seeded with 20 imported infections on 1 March 2022 and run forward for 6 months. We consider five and ten seeds as sensitivity analyses (Supplementary Fig. 2). Upon infection with SARS-CoV-2, susceptible individuals (S) enter an exposed (latent) compartment (L) before becoming infectious. We consider that children and adolescents were less susceptible to infection compared with adults^44,45^. A sensitivity analysis considering homogeneous susceptibility across age groups is presented in Supplementary Fig. 3. Exposed individuals stay in their compartment for an average of 1/*γ*_E_ = 1.2 days before moving to either asymptomatic (I_A_) or symptomatic (I_S_) compartments according to the age-specific probability of being asymptomatic ($1 - P_a^s$). No difference in infectiousness between asymptomatic and symptomatic individuals was considered in the main analyses^46^, whereas asymptomatic individuals were considered to be 65% less infectious than symptomatic ones in a sensitivity analysis^47^ (Supplementary Fig. 4). An age-dependent proportion ($P_a^h$) of symptomatic cases require hospitalization (H), while the rest of symptomatic cases and all asymptomatic infections recover naturally (R) (Extended Data Fig. 1a). We assume asymptomatic infections and nonhospitalized symptomatic cases to stay in their compartments for an average of 1/*γ*_I_ = 5.6 days, thus resulting in mean intrinsic generation time of 6.8 days, as previously estimated for Omicron^48^ (Supplementary Table 5).

For patients requiring hospitalization (H), the average time from symptom onset to hospital admission was 1/*γ*_SH_ = 2.2 days (ref. ^49^). We assume that hospitalized patients do not transmit the virus. We divided the hospital settings (H) into two parts: the general ward (Hosp) and ICU ward (ICU), as illustrated in Extended Data Fig. 1b. Once admitted to hospital, a patient either remains in the general ward until discharge or is transferred to an ICU according to an age-dependent ICU admission risk. We assume that patients admitted to an ICU entered the ICU on the same day they were admitted to hospital. Patients in the general ward (or ICU) could either stay in the general ward (or ICU) until they are discharged or die, based on the corresponding mortality risk. We assume that all deaths occur among hospitalized patients.

To capture the potential impact of newly available antiviral therapies, we divided symptomatic cases (I_S_) into two categories: those who timely received an antiviral therapy after symptom onset, and those who did not (Extended Data Fig. 1c).

All compartments and transition flows are duplicated into parallel branches that represent primary (V) and booster (B) vaccinations (Extended Data Fig. 1d). We assume that only susceptible individuals in compartment S are eligible for primary vaccination. To describe the recommended two-dose primary vaccination (common to the two inactivated vaccines currently widely used in China: Sinovac/CoronaVac and Sinopharm/BBIBP-CorV), compartment V was further stratified into two vaccination strata (V_1_ and V_2_), differentiating individuals who have received one or two doses, respectively. Only uninfected individuals who have completed their primary vaccination schedules by 6 months (1/*ω*_P_) will receive a booster shot (B). Each dose produces a vaccine protection (V_1_^e^, V_2_^e^, B^e^) after an average of 14 days (1/*ω*_1_, 1/*ω*_3_ and 1/*ω*_4_).

We model VE against infection using a ‘leaky’ vaccine in which all vaccinated individuals are exposed to a lower risk of infection, which is 1 – VE times that of nonvaccinated individuals^50^. Like vaccination-induced protection, infection-induced immunity wanes over time (Extended Data Fig. 1d). After an average of 180 days (1/*ω*_P_) since the second dose, primary-vaccinated individuals move to a new compartment (‘waned vaccine effectiveness’, V_2_W); individuals in this compartment are ready for receiving their booster shots. Likewise, 180 days (1/*ω*_B_) after the booster shot, boosted individuals move to a new compartment (‘waned vaccine effectiveness’, BW). Waning of infection-induced immunity acts in a manner different from that of the vaccine. Individuals who have recovered from SARS-CoV-2 infection (R) are protected against reinfection with the same variant for an exponentially distributed duration with mean 1/*ω*_R_ days, after which they move back to the susceptible compartment. The transition rates for vaccine- and infection-induced immunity processes are defined in Supplementary Table 6 (ref. ^51^). The VEs against different clinical endpoints in the different stages of vaccine protection are reported in Supplementary Table 1. Details are reported in Vaccine effectiveness.

### Model of COVID-19 burden

#### Age-specific risks

To measure the burden (that is, hospitalizations, ICU admissions and deaths) and the strain of the healthcare system, we rely on the age-specific infection fatality risk (IFR) and infection hospitalization risk among unvaccinated individuals from the Omicron wave in Hong Kong SAR, China^39^. The Hong Kong Center for Health Protection publishes reported case fatality ratios by age group and vaccination status^52^, and compares the age profiles of reported cases against resident population^53^. Since the age profile of cumulative reported cases is very similar to the resident population by the end of 2021 in Hong Kong SAR, we assume the undetected infections have the same age profile as the reported cases. We thus estimate the age-specific IFR by dividing the age-specific case fatality ratios among unvaccinated individuals by the overall infection-reporting ratio. A modeling report on the fifth wave of COVID-19 in Hong Kong SAR^54^ estimates that around 4.5 million residents of Hong Kong had been infected by 21 April 2022, as of which day 1.18 million cumulative case were reported. We further calculate age-specific infection hospitalization risk from the Hong Kong Omicron wave^39^ by dividing the estimated IFR by the age-specific fatality risk among hospitalized patients who were not fully vaccinated (B. J. Cowling, personal communication, 2022) (Supplementary Table 7).

For the age-specific ICU admission risk of hospitalized patients, we adjusted the ICU admission risk associated with the ancestral lineage reported in China^55^, by the ratio of the overall ICU admission risk among unvaccinated hospitalized patients infected with the Omicron variant (19.0% (ref. ^56^)) and those infected with the ancestral lineage (6.4% (ref. ^55^)). The estimated age-specific risks of disease progression are presented in Supplementary Tables 7 and 8 (ref. ^57^).

#### Duration of hospital and ICU stay

We set the length of stay in hospital to 6 days; 8 days are considered for non-ICU hospitalizations with fatal outcomes based on observations in the Hong Kong Omicron wave^39^ (B. J. Cowling, personal communication, 2022). We assumed the ICU length of stay to be 8 days, consistent with literature reports^58,59^.

#### Healthcare resources

As of 2020, a total of 9.1 million hospital beds were available in China. Among them, 3.14 million were reserved for respiratory illness (including hospital beds in departments of internal medicine, pediatrics, infectious disease and ICUs), 64,000 of which are ICUs^31^.

### Model validation against the Omicron outbreak in Shanghai

We calibrated the transmissibility and proportion of symptomatic cases to the field data of the Omicron BA.2 variant outbreak in Shanghai, China. We used a Bayesian approach^60^ to estimate the net reproduction number *R*_*t*_ for the initial phase (from 1 March to 8 March 2022) of the epidemic in Shanghai, before strict control measures were implemented. The method is based on the analysis of the epidemic curve of symptomatic cases and on the knowledge of the generation time, which is assumed to be Gamma distributed with mean 6.8 days (shape = 2.39, scale = 2.95) as estimated for the Omicron variant in a previous study^48^. The resulting estimate of the average reproduction number *R*_*t*_ is 3.4. We then follow the approach in Marziano et al.^61^ based on the next generation matrix to calculate the model transmission rate from the estimated reproduction number while accounting for the vaccine-induced partial protection of the population. When removing the effect of vaccination, we estimated the reproduction number *R* of the Omicron BA.2 variant to be 3.9 at the beginning of the 2022 outbreak in Shanghai. Such an estimate is conditional on the situation on the ground at the beginning of March 2022, where, although no strict NPIs were implemented, a mask mandate was still in place and the behavior of the population may have been different from prepandemic standards.

City-wide screenings are being conducted frequently throughout the course of the Shanghai outbreak allowing the identification of most infected individuals, regardless of the presence/absence of symptoms. Therefore, to estimate the association between symptoms and infection, we simulated our compartmental model for the population of Shanghai (which also considers city-specific vaccination rates) assuming *R*_*t*_ to be 3.4. We then modulated the age-specific probability of developing symptoms^62^ by a scaling factor that is chosen to fit both the curves of symptomatic and asymptomatic infections in Shanghai between 1 March and 8 April 2022 (Extended Data Fig. 2). We further adjusted the calibrated age-specific probability of developing symptoms by the ratio of the proportion of confirmed cases among total infections observed in Shanghai during the initial phase (from 1 March to 8 April 2022) (3.50%) to that from 1 March to 28 April 2022 (9.24%)^63^.

Finally, as a sensitivity analysis, we also calibrated the model using a shorter generation time, in line with estimates for the Delta variant in the United Kingdom (4.7 days; ref. ^64^) (Supplementary Fig. 5).

### Mitigation with vaccination

A mass vaccination campaign has been launched in China since December 2020 (ref. ^65^). On 3 October 2021, a homologous booster vaccination campaign (relying on the same vaccine as the initial inactivated vaccine shots) has been initiated among individuals aged ≥18 years who completed primary vaccination at least 6 months earlier^66,67^. As of 12 April 2022, >90% of populations aged ≥3 years have completed primary vaccination and >50% of the populations has received a booster dose^68^. Compared with other age groups (86.4%, 100% and 92.3% fully vaccinated individuals for the age groups 3–11, 12–17 and 18–59 years, respectively), individuals aged ≥60 years have the lowest vaccination coverage (about 80%)^69^, corresponding to approximately 52 million unvaccinated individuals^28^.

From 1 March 2022 onwards, homologous booster rollout was set at 5 million doses per day in the baseline analysis. Sensitivity analyses on heterologous booster vaccination using subunit, mRNA, and vector vaccines were conducted (Supplementary Fig. 6). The only difference between heterologous and homologous booster considered in the model is VE (values reported in Supplementary Table 1). Vaccine coverage over time and by age group for the baseline scenario is presented in Supplementary Fig. 7.

#### Vaccine effectiveness

We considered different VEs against different clinical endpoints (namely, infection, symptomatic illness, hospitalization and death) and onward transmission. As shown in Supplementary Table 1, VEs against these clinical outcomes at the following five time points are considered: 14 days after receiving the first dose, 14 days after receiving the second dose, 14 days after the booster dose, 6 months after the second dose of primary 2-dose vaccination (2W) and 6 months after the booster dose (BW). To account for the decay of VEs, either 6 months after the second dose of primary vaccination or after the booster dose, vaccinated individuals move to the ‘waned vaccine effectiveness’ compartments V_2_W and BW, respectively.

VEs against symptomatic disease, hospitalization and death after receiving two doses of inactivated vaccines, a homologous booster using inactivated vaccines and a heterologous booster using mRNA vaccines were estimated during the Omicron waves in Hong Kong or Brazil^27,32^. For other VEs without field estimates, we used a verified statistical model^70^ to predict vaccine protection based on the levels of neutralizing antibody titers (NATs) against Omicron between different booster regimens and time points summarized in Supplementary Table 9 (refs. ^71–74^). The Omicron variant shows very high immune escape potential. Peiris et al.^75^ found a 6.4-fold and 9.7-fold decrease in the level of NATs against Omicron 6 months after administering two doses and 1 month after administering a booster dose of inactivated vaccines, respectively, compared with those against the ancestral lineage (low immune escape scenario). We further conducted a sensitivity analysis for a high immune escape scenario, which considers a 19.1-fold decrease in the NAT against Omicron both after administering two doses and a booster dose of inactivated vaccines, compared with that against the ancestral lineage^76^. For VEs against symptomatic illness, hospitalization, and death, we use conditional VEs, which are calculated according to the formulas presented in Supplementary Table 10.

### Mitigation through antiviral therapies

A homegrown monoclonal neutralizing antibody therapy (BRII-196/BRII-198 combination) and an imported antiviral therapy (nirmatrelvir tablet/ritonavir tablet combination) have been approved for emergency use in China^25,26^. In the baseline scenario, we do not consider antiviral therapies. To quantify the mitigating effect of antiviral therapies, we simulated two alternative scenarios: (1) 50% of symptomatic cases will receive an antiviral therapy with an efficacy of 80% in preventing hospitalizations and deaths (in agreement with the estimate for the Chinese manufactured BRII-196/BRII-198 combination)^33^, and (2) 100% of symptomatic cases will receive an antiviral therapy with an efficacy of 89% in preventing hospitalizations and deaths (in agreement with the estimate for the imported nirmatrelvir tablet/ritonavir tablet combination^34,77^). Only symptomatic cases of patients aged ≥12 years are eligible to receive COVID-19 antiviral therapies^25^ (Supplementary Table 6). The rationale for the 50% treatment uptake scenario is that not all symptomatic cases may be promptly identified, thus leading either to receive the treatment well after symptom onset (and thus the effectiveness of the antiviral therapy is reduced) or to entirely missing potential eligible individuals. The 100% treatment uptake scenario represents an ideal scenario where all eligible symptomatic infections receive the treatment at the peak of its efficacy.

### Mitigation through NPIs

We tested the impact of NPIs in two ways: (1) implementing a national school closure or a national school and workplace closure by removing contacts that occurred in schools or workplaces from the baseline mixing patterns (Supplementary Fig. 1) and (2) reducing effective contacts equally across age groups, which is modeled as a reduction in the reproduction number; specifically, we considered *R*_*t*_ = 2.0, 2.5, 3.0 and 3.9 that represent varying intensities of NPIs (Supplementary Table 5).

### Geographical heterogeneity across the Chinese population

To account for within-China heterogeneity, we run the baseline analysis for three other highly diverse contexts: (1) an urbanized setting with a relatively low vaccine coverage (Shanghai), (2) a rural setting with a relatively low vaccine coverage (Shanxi, a central province in China) and (3) a high vaccination setting (Shandong, an eastern coastal province). In our analysis, these settings differ in terms of primary and booster vaccination coverages, age structure of the population, contact patterns of the population^42^, number of available hospital beds and ICUs^31^ (Supplementary Table 2 and Supplementary Fig. 1). The overall and age-specific incidence rates of different clinical endpoints over a 6-month simulated period across the three settings are compared with the ‘national average’.

### Statistical analysis

For each scenario, we performed 200 stochastic simulations. The outcomes of these simulations determined the distribution of the number of symptomatic infections, hospital admissions, ICU admissions and deaths by age. We defined 95% credible intervals as quantiles 0.025 and 0.975 of the estimated distributions.

### Reporting summary

Further information on research design is available in the Nature Research Reporting summary linked to this article.

## Online content

Any methods, additional references, Nature Research reporting summaries, source data, extended data, supplementary information, acknowledgements, peer review information; details of author contributions and competing interests; and statements of data and code availability are available at 10.1038/s41591-022-01855-7.

## Supplementary information

Supplementary Information Supplementary Tables 1–10 and Figs. 1–7.

Reporting Summary

## Data Availability

The data used in the study are provided in Supplementary Information and are available with the code on GitHub at https://github.com/DXW-sola1015/Model_Omicron_China.

## Code availability

The codes used in this study are available on GitHub at https://github.com/DXW-sola1015/Model_Omicron_China.
