# Supplementary material for: Modeling transmission of SARS-CoV-2 Omicron in China
Source: Nat Med. 2022 May 10;28(7):1468–75. doi: 10.1038/s41591-022-01855-7 (PMC9307473; doi:10.1038/s41591-022-01855-7)
Supplement: Supplementary file 1 — Supplementary Tables 1–10 and Figs. 1–7. [file 41591_2022_1855_MOESM1_ESM.pdf]

nature portfolio

https://doi.org/10.1038/s41591-022-01855-7

Supplementary information

Modeling transmission of SARS-CoV-2 Omicron in China

In the format provided by the authors and unedited

Modeling transmission of SARS-CoV-2 Omicron in China

Jun Cai[1]*, Xiaowei Deng[1]*, Juan Yang[1,2]*, Kaiyuan Sun[3], Hengcong Liu[1], Zhiyuan Chen[1], Cheng Peng[1], Xinhua Chen[1], Qianhui Wu[1], Junyi Zou[1], Ruijia Sun[1], Wen Zheng[1], Zeyao Zhao[1], Wanying Lu[1], Yuxia Liang[1], Xiaoyu Zhou[1], Marco Ajelli[4†], Hongjie Yu[1,2†]

1. School of Public Health, Fudan University, Key Laboratory of Public Health Safety, Ministry of Education, Shanghai, China
2. Shanghai Institute of Infectious Disease and Biosecurity, Fudan University, Shanghai, China
3. Division of International Epidemiology and Population Studies, Fogarty International Center, National Institutes of Health, Bethesda, MD, USA
4. Laboratory for Computational Epidemiology and Public Health, Department of Epidemiology and Biostatistics, Indiana University School of Public Health, Bloomington, IN, USA

*These authors contributed equally to this work.

[†]These authors are joint senior authors contributed equally to this work.

Corresponding authors: Hongjie Yu, School of Public Health, Fudan University, Key Laboratory of Public Health Safety, Ministry of Education, Shanghai 200032, China

E-mail: yhj@fudan.edu.cn

Supplementary Tables

Supplementary Table 1 | Vaccine efficacy assumptions. Overall vaccine efficacy (%) against infection, symptomatic disease, hospitalization, and mortality, and conditional vaccine efficacy against onward transmission given breakthrough infection for the Omicron variant (low immune escape scenario vs. high immune escape scenario listed separately) and for inactivated vaccines primary course, homologous and heterologous boosters in the model. 1 = one dose; 2 = two doses; 2W = 6 months waned from two doses; B = booster doses; BW = 6 months waned from booster doses. Values in bold are real-world effectiveness estimated from the Omicron waves in Hong Kong[27] or Brazil[32].

**Immune escape**	**Outcome**	**Inactivated primary**			**Inactivated booster**		**mRNA booster**		**Subunit booster**		**Vector booster**	
		1	2	2W	B	BW	B	BW	B	BW	B	BW
Low (optimistic)	Infection	5.6	9.1	5.9	17.0	13.8	53.2	43.1	41.0	33.2	52.7	42.7
	Symptomatic disease	**16.5**	**26.9**	17.3	**46.5**	37.8	56.8	46.2	57.4	46.7	57.0	46.3
	Hospitalization	**46.2**	**78.8**	69.4	**98.1**	93.1	98.1	93.1	98.1	93.1	98.1	93.1
	Mortality	**56.3**	**83.2**	75.0	**98.4**	95.1	98.4	95.1	98.4	95.1	98.4	95.1
	Onward transmission	0	0	0	10.6	0	22	0	10.6	0	10.6	0
High (pessimistic)	Infection	0.6	4.8	3.1	9.2	7.7	28.1	23.4	21.6	18.0	27.8	23.2
	Symptomatic disease	2.1	**17.9**	11.5	**18.0**	14.6	32.5	26.4	32.9	26.7	32.6	26.5
	Hospitalization	44.0	**62.6**	55.2	**71.3**	67.6	**85.5**	81.0	87.3	82.7	88.2	83.6
	Mortality	53.0	**66.3**	59.7	**79.2**	76.3	**87.0**	83.8	87.5	84.3	88.8	85.6
	Onward transmission	0	0	0	0	0	0	0	0	0	0	0

Supplementary Table 2 | Age structure of population (in 2020), vaccination coverage (as of March 2022) and number of available hospital beds and ICUs (in 2020) across four settings

Setting	Age group	Population	Primary coverage (%)	Booster coverage (%)	Hospital (non-ICU) beds	ICU beds
**Shanghai**	0-2	514500	0.0	0.0	-	-
	3-11	1522920	72.6	0.0	-	-
	12-17	825000	100.0	0.0	-	-
	18-59	16193080	77.4	54.7	-	-
	60-69	3414100	72.6	43.9	-	-
	70+	2401500	46.7	28.4	-	-
	overall	24871100	72.7	44.4	51109	815
**Shanxi**	0-2	993998	0.0	0.0	-	-
	3-11	3182816	83.3	0.0	-	-
	12-17	2569457	100.0	0.0	-	-
	18-59	23068541	88.8	34.3	-	-
	60-69	3162930	82.0	37.6	-	-
	70+	1937875	68.3	26.4	-	-
	overall	34915617	84.8	27.5	64048	1091
**Shandong**	0-2	3993036	0.0	0.0	-	-
	3-11	11449183	84.7	0.0	-	-
	12-17	6634307	100	0.0	-	-
	18-59	58230121	96.9	74.4	-	-
	60-69	10983372	91.6	74.5	-	-
	70+	10237434	86.5	70.3	-	-
	overall	101527453	90.3	57.8	162047	5624
**China**	0-2	46730333	0.0	0.0	-	-
	3-11	155500009	88.0	0.0	-	-
	12-17	94764080	100.0	0.0	-	-
	18-59	848766084	93.8	51.5	-	-
	60-69	147388498	86.6	56.4	-	-
	70+	116629720	72.2	39.6	-	-
	overall	1409778724	87.9	46.0	3080364	63527

Supplementary Table 3 | Description of the compartments of SARS-CoV-2 transmission and vaccination model

Parameter	Description
N_a	Number of individuals in age group *a*
S_a	Number of unvaccinated individuals who are fully susceptible to SARS-CoV-2 infection in age group *a*
E_a	Number of latent unvaccinated individuals in age group *a*
$I_{S,a}$	Number of infectious unvaccinated symptomatic cases, including both $I_{S,a}^{drug}$ and $I_{S,a}^{naive}$, in age group *a*
$I_{S,a}^{drug}$	Number of infectious unvaccinated symptomatic cases who will take a COVID-19 drug in age group *a*
$I_{S,a}^{naive}$	Number of infectious unvaccinated symptomatic cases who will not take a COVID-19 drug in age group *a*
$I_{A,a}$	Number of infectious unvaccinated asymptomatic individuals in age group *a*
H_a	Number of unvaccinated hospitalized patients, including both $Hosp_a$ and ICU_a, in age group *a*
$Hosp_a$	Number of unvaccinated hospitalized (non-ICU) patients in age group *a*
ICU_a	Number of unvaccinated ICU patients in age group *a*
D_a	Number of unvaccinated deaths in age group *a*
R_a	Number of unvaccinated individuals who recovered from infection in age group *a*
$V_{1,a}$	Number of individuals vaccinated the first dose of two-dose primary series in age group *a*, for whom the first dose is not effective yet
$V_{1,a}^e$	Number of individuals vaccinated the first dose in age group *a*, for whom the first dose is effective
$V_{2,a}$	Number of individuals vaccinated the second dose of two-dose primary series in age group *a*, for whom the second dose is not effective yet
$V_{2,a}^e$	Number of individuals vaccinated the second dose in age group *a*, for whom the second dose is effective
$V_{2W,a}$	Number of individuals vaccinated the second dose in age group *a*, for whom the primary vaccination protection has waned for six months
$V_{B,a}$	Number of individuals vaccinated the booster dose in age group *a*, for whom the booster dose is not effective yet
$V_{B,a}^e$	Number of individuals vaccinated the booster dose in age group *a*, for whom the booster dose is effective
$V_{BW,a}$	Number of individuals vaccinated the booster dose in age group *a*, for whom the booster vaccination protection has waned for six months
R_a^V	Number of vaccinated individuals who recovered from infection in age group *a*

Supplementary Table 4 | Description of model parameters

Parameter	Description
n	The number of age groups
**Epidemiology**	
$\lambda_a(t)$	Force of infection for age group *a* at time *t*
β	Transmission rate in the absence of NPIs, inferred from the value of the reproduction number *R* for the SARS-CoV-2 Omicron variant
φ	Reduction of the transmission rate due to NPIs
r_a	Susceptibility to SARS-CoV-2 infection in age group *a*
$M_{a,\tilde{a}}$	Mean number of daily contacts that an individual in age group *a* has with individuals in age group $\tilde{a}$
$1/\gamma_E$	Average duration of latent period (days)
$1/\gamma_I$	Average infectious periods (days)
κ	Infectivity of an asymptomatic individual relative to a symptomatic individual
**Disease burden**	
$1/\gamma_{SH}$	Average time from symptom onset to hospitalization (days)
$1/\gamma_{HD}$	Average time from hospital (non-ICU) admission to death (days)
$1/\gamma_{UD}$	Average time from ICU admission to death (days)
$1/\gamma_{HR}$	Length of hospital stay before recovery (days)
$1/\gamma_{UR}$	Length of ICU stay before recovery (days)
p_a^s	Age-dependent proportion of unvaccinated infections who developed symptoms
p_a^h	Age-dependent proportion of unvaccinated symptomatic infections requiring hospitalizations
p_a^{HD}	Age-dependent fatality rate among unvaccinated hospitalized (non-ICU) patients
p_a^{UD}	Age-dependent fatality rate among unvaccinated ICU patients
p_a^{icu}	Age-dependent proportion of unvaccinated hospitalized patients requiring ICU
**Primary vaccination**	
$\alpha_{1,a}(t)$	Vaccination rate (first dose) for age group *a* at time *t*
$1/\omega_1$	Average interval between administration of the first dose and full protection by the first dose (days)
ϵ_1^{inf}	Overall VE against infection after the first dose is effective
ϵ_1^{symp}	Overall VE against symptomatic disease after the first dose is effective
ϵ_1^{hosp}	Overall VE against hospitalization after the first dose is effective
ϵ_1^{death}	Overall VE against mortality after the first dose is effective
ϵ_1^{tran}	Conditional VE against onward transmission given infection after the first dose is effective
$\epsilon_1^{symp\|inf}$	Conditional VE against symptomatic disease given infection after the first dose is effective
$\epsilon_1^{hosp\|symp}$	Conditional VE against hospitalization given symptom after the first dose is effective
$\epsilon_1^{death\|hosp}$	Conditional VE against death given hospitalization after the first dose is effective
$1/\omega_2$	Average interval between full protection of the first dose and administration of the second dose (days)
$\alpha_{2,a}(t)$	Vaccination rate (second dose) for age group *a* at time *t*
$1/\omega_3$	Average interval between administration of the second dose and full protection by the second dose (days)
ϵ_2^{inf}	Overall VE against infection after the second dose is effective
ϵ_2^{symp}	Overall VE against symptomatic disease after the second dose is effective

ϵ_2^{hosp}	Overall VE against hospitalization after the second dose is effective
ϵ_2^{death}	Overall VE against mortality after the second dose is effective
ϵ_2^{tran}	Conditional VE against onward transmission given infection after the second dose is effective
$\epsilon_2^{symp\|inf}$	Conditional VE against symptomatic disease given infection after the second dose is effective
$\epsilon_2^{hosp\|symp}$	Conditional VE against hospitalization given symptom after the second dose is effective
$\epsilon_2^{death\|hosp}$	Conditional VE against death given hospitalization after the second dose is effective
**Waned primary vaccination**	
$1/\omega_P$	Average interval between full protection of the second dose and administration of the booster dose (days)
ϵ_{2W}^{inf}	Overall VE against infection wanned after the second dose is effective
ϵ_{2W}^{symp}	Overall VE against symptomatic disease waned after the second dose is effective
ϵ_{2W}^{hosp}	Overall VE against hospitalization waned after the second dose is effective
ϵ_{2W}^{death}	Overall VE against mortality waned after the second dose is effective
ϵ_{2W}^{tran}	Conditional VE against onward transmission given infection waned after the second dose is effective
$\epsilon_{2W}^{symp\|inf}$	Conditional VE against symptomatic disease given infection waned after the second dose is effective
$\epsilon_{2W}^{hosp\|symp}$	Conditional VE against hospitalization given symptom waned after the second dose is effective
$\epsilon_{2W}^{death\|hosp}$	Conditional VE against death given hospitalization waned after the second dose is effective
**Booster vaccination**	
$\alpha_{VB,a}(t)$	Vaccination rate (booster dose) for age group *a* at time *t*
$1/\omega_4$	Average interval between administration of the booster dose and full protection by the booster dose (days)
ϵ_B^{inf}	Overall VE against infection after the booster dose is effective
ϵ_B^{symp}	Overall VE against symptomatic disease after the booster dose is effective
ϵ_B^{hosp}	Overall VE against hospitalization after the booster dose is effective
ϵ_B^{death}	Overall VE against death after the booster dose is effective
ϵ_B^{tran}	Conditional VE against onward transmission given infection after the booster dose is effective
$\epsilon_B^{symp\|inf}$	Conditional VE against symptomatic disease given infection after the booster dose is effective
$\epsilon_B^{hosp\|symp}$	Conditional VE against hospitalization given symptom after the booster dose is effective
$\epsilon_B^{death\|hosp}$	Conditional VE against death given hospitalization after the booster dose is effective
**Waned booster vaccination**	
$1/\omega_B$	Average interval between full protection of the booster dose and waned booster protection (days)
ϵ_{BW}^{inf}	Overall VE against infection wanned after the booster dose is effective
ϵ_{BW}^{symp}	Overall VE against symptomatic disease waned after the booster dose is effective
ϵ_{BW}^{hosp}	Overall VE against hospitalization waned after the booster dose is effective
ϵ_{BW}^{death}	Overall VE against mortality waned after the booster dose is effective
ϵ_{BW}^{tran}	Conditional VE against onward transmission given infection waned after the booster dose is effective
$\epsilon_{BW}^{symp\|inf}$	Conditional VE against symptomatic disease given infection waned after the booster dose is effective
$\epsilon_{BW}^{hosp\|symp}$	Conditional VE against hospitalization given symptom waned after the booster dose is effective
$\epsilon_B^{death\|hosp}$	Conditional VE against death given hospitalization waned after the booster dose is effective

**Protection from prior infection**	
$1/\omega_R$	Average duration that the protection gained from prior infection lasts (days)
$\epsilon_R^{symp\|inf}$	Conditional protection against symptomatic disease given infection after recovery
$\epsilon_R^{hosp\|symp}$	Conditional protection against hospitalization given symptom after recovery
$\epsilon_R^{death\|hosp}$	Conditional protection against death given hospitalization after recovery
**COVID-19 drug**	
p_a^{drug}	Age-dependent proportion of symptomatic cases taking a COVID-19 drug
ϵ^{drug}	Protection against hospitalization or death for a symptomatic case taking a COVID-19 drug

Supplementary Table 5 | Summary of epidemiological parameter values

Parameter	Value	Sensitivity analysis
R	3.9	
φ	0	0.231, 0.359, and 0.487 for R_t = 3.0, 2.5, and 2.0 by implementing varying intensities of NPIs
r_a	$r_a = 0.56$ (95% CI: 0.37–0.85) when $a < 20$; $r_a = 1$ when $a \geq 20$ [44,45]	$r_a = 1$ for all age groups (homogeneous susceptibility)
$M_{a,\tilde{a}}$	Contact matrix in China before the pandemic[42] (see Supplementary Fig. 1a)	Synthetic contact matrix for school closure in China (see Supplementary Fig. 1b); Synthetic contact matrix for school and workplace closure in China (see Supplementary Fig. 1c); Contact matrix in Shandong before the pandemic[42] (see Supplementary Fig. 1d); Contact matrix in Shanghai before the pandemic[42] (see Supplementary Fig. 1e); Contact matrix in Shanxi before the pandemic[42] (see Supplementary Fig. 1f)
$1/\gamma_E$	1.2 [64]	
$1/\gamma_I$	5.64 [48]	3.5 [64]
κ	1 [46]	0.35 [47]

Supplementary Table 6 | Summary of vaccine and drug relevant parameter values

Parameter	Value	Sensitivity analysis
p_a^{drug}	0% for adults and adolescents aged ≥12 years	50%, 100%
ϵ^{drug}	80% for adults and adolescents aged ≥12 years (BRII-196/BRII-198 combination therapy)[33]	89% for adults and adolescents aged ≥12 years (nirmatrelvir tablet/ritonavir tablet combination therapy)[34,77]
$\alpha_{1,a}(t)$	See Supplementary Fig. 7	
$\alpha_{2,a}(t)$	See Supplementary Fig. 7	
$\alpha_{VB,a}(t)$	5 million doses per day (see Supplementary Fig. 7)	
$1/\omega_1$	14	
$1/\omega_2$	7	
$1/\omega_3$	14	
$1/\omega_P$	180	
$1/\omega_4$	14	
$1/\omega_B$	180	
$1/\omega_R$	900 [51]	

Supplementary Table 7 | Summary of parameter values used for inferring those on disease progression in the mathematical model

Parameter	Description	Value	Source
p_a^{CFR}	Age-specific case fatality risk (CFR) among unvaccinated individuals infected with the Omicron variant	Age 0–2: 0.008%<br>Age 3–11: 0.011%<br>Age 12–19: 0.043%<br>Age 20–29: 0.039%<br>Age 30–39: 0.058%<br>Age 40–49: 0.212%<br>Age 50–59: 0.868%<br>Age 60–69: 2.080%<br>Age 70–79: 5.527%<br>Age ≥80 years: 16.482%	Estimates from the Hong Kong Omicron wave[52]
p_a^{HFR}	Age-specific hospitalization fatality risk (HFR) among unvaccinated patients infected with the Omicron variant	Age 0–19: 0.21%<br>Age 20–29: 1.64%<br>Age 30–44: 1.78%<br>Age 45–64: 11.12%<br>Age 65–79: 21.27%<br>Age ≥80 years: 43.16%	Estimates from the Hong Kong Omicron wave (B. J. Cowling, personal communication, 2022)
f_a	Age-specific ratio between ICU death risk and in-hospital death risk for the wild-type reported in France	Age 0–39: 13.0<br>Age 40–49: 6.4<br>Age 50–59: 5.8<br>Age 60–69: 4.2<br>Age 70–69: 2.6<br>Age ≥80 years: 1.5	[59]
$p_{a,WT}^{icu}$	Age-specific ICU admission risk of hospitalized patients infected with the wild-type reported in China	Age 0–14: 0.000%<br>Age 15–49: 2.154%<br>Age 50–64: 7.192%<br>Age ≥65 years: 20.915%<br>Overall: 6.43%	[55,57]
$\Delta_{O\to WT}^{icu}$	Risk ratio of ICU admission for unvaccinated hospitalized patients with the Omicron variant compared to the wild-type	19.0%/6.43%=2.9	[15,55,56]
$p_{a,WT}^{s}$	Age-specific proportion of infections who developed symptoms for the wild-type reported in Italy	Age 0–19: 18.09%<br>Age 20–39: 22.41%<br>Age 40–59: 30.54%<br>Age 60–79: 35.46%<br>Age ≥80 years: 64.56%<br>Overall: 31.02%	[62]
$\Delta_{O\to WT}^{s}$	Ratio of probability of developing symptoms among unvaccinated infections with the Omicron variant compared to the wild-type	0.15	Calibrated against the Omicron outbreak in Shanghai

Supplementary Table 8 | Summary of parameter values on disease progression

Parameter	Value
$1/\gamma_{SH}$	2.2 [49]
$1/\gamma_{HD}$	8 (estimated from the Hong Kong Omicron wave; B. J. Cowling, personal communication, 2022)
$1/\gamma_{UD}$	8 [58]
$1/\gamma_{HR}$	6 (estimated from the Hong Kong Omicron wave; B. J. Cowling, personal communication, 2022)
$1/\gamma_{UR}$	8 [58]
p_a^s	Age 0–17: 7.16%; Age 18–39: 8.87%; Age 40–59: 12.09%; Age ≥60 years: 15.89%
p_a^h	Age 0–2: 11.09%; Age 3–11: 6.37%; Age 12–17: 15.01%; Age 18–29: 7.41%;<br>Age 30–39: 9.78%; Age 40–49: 8.02%; Age 50–59: 17.06%; Age 60–69: 20.45%;<br>Age ≥70 years: 65.60%
p_a^{HD}	Age 0–2: 0.26%; Age 3–11: 0.52%; Age 12–17: 0.75%; Age 18–29: 0.96%;<br>Age 30–39: 0.80%; Age 40–49: 3.23%; Age 50–59: 5.75%; Age 60–69: 8.68%;<br>Age ≥70 years: 24.98%
p_a^{UD}	Age 0–2: 3.40%; Age 3–11: 6.74%; Age 12–17: 9.81%; Age 18–29: 12.48%;<br>Age 30–39: 10.45%; Age 40–49: 20.66%; Age 50–59: 33.32%; Age 60–69: 36.45%;<br>Age ≥70 years: 37.47%
p_a^{icu}	Age 0–2: 0.23%; Age 3–11: 1.61%; Age 12–17: 3.33%; Age 18–29: 5.39%;<br>Age 30–39: 9.82%; Age 40–49: 14.66%; Age 50–59: 19.49%; Age 60–69: 29.67%;<br>Age ≥70 years: 48.22%

Supplementary Table 9 | Parameters used in the efficacy prediction model

**Vaccines**	**Neutralizing antibody level against ancestral lineage after a homologous/heterologous booster**	**Fold-reduction of neutralizing antibody level (Omicron vs. prototype)**	**Neutralizing antibody level of convalescents' serum**
2 doses of CoronaVac + 1 dose of CoronaVac[10,71,72,75]	28 days after primary two doses: 49.1<br>180 days after primary two doses: 6.7<br>28 days after booster doses: 143.3<br>180 days after booster doses: 36.4	After primary two doses: 6.4<br>After booster dose: 9.7	163.7
2 doses of CoronaVac + 1 dose of ZF001[73]	14 days after booster doses: 1305.70	7.26	324.7
2 doses of CoronaVac + 1 dose of Pfizer[74,75]	28 days after booster doses: 186.21	7.30	58.8*
	1 month after booster doses: 320.00	6.40	120.64*

* Adjusted convalescent serum titers were used.

Supplementary Table 10 | Conditional vaccine efficacy against different clinical outcomes

VE against	Calculation
Symptoms given infection	$\epsilon^{symp\|inf} = \dfrac{\epsilon^{symp} - \epsilon^{inf}}{1 - \epsilon^{inf}}$
Hospitalization given symptoms	$\epsilon^{hosp\|symp} = \dfrac{\epsilon^{hosp} - \epsilon^{symp}}{1 - \epsilon^{symp}}$
Death given hospitalization	$\epsilon^{death\|hosp} = \dfrac{\epsilon^{death} - \epsilon^{hosp}}{1 - \epsilon^{hosp}}$

Supplementary Figures

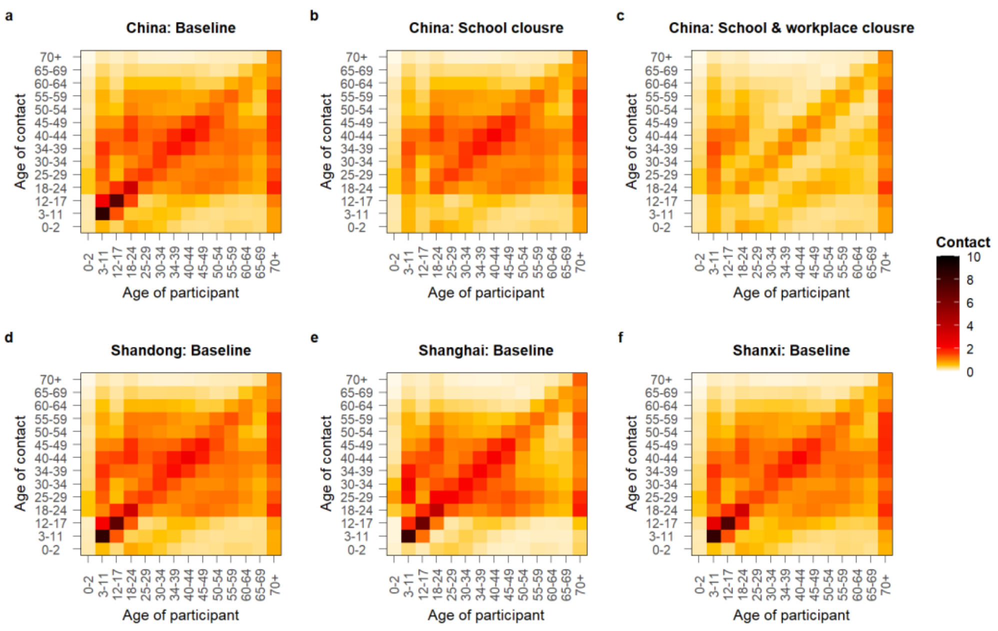

Supplementary Fig. 1 | Age-mixing patterns in four settings. a, Pre-pandemic contact matrix in China. **b**, Contact matrix removing contacts occurred in schools in China. **c**, Contact matrix removing contacts occurred in schools and workplaces in China. **d**, Pre-pandemic contact matrix in Shandong. **e**, Pre-pandemic contact matrix in Shanxi. **f**, Pre-pandemic contact matrix in Shanghai. The contact matrix in panel **a** refers to the baseline scenario. The country-level mixing patterns were generated using the contact survey data in Shanghai in 2017–2018[42]. Each cell of the matrix represents the mean number of daily contacts that an individual in a given age group has with other individuals, stratified by age group. The color intensity represents the number of daily contacts.

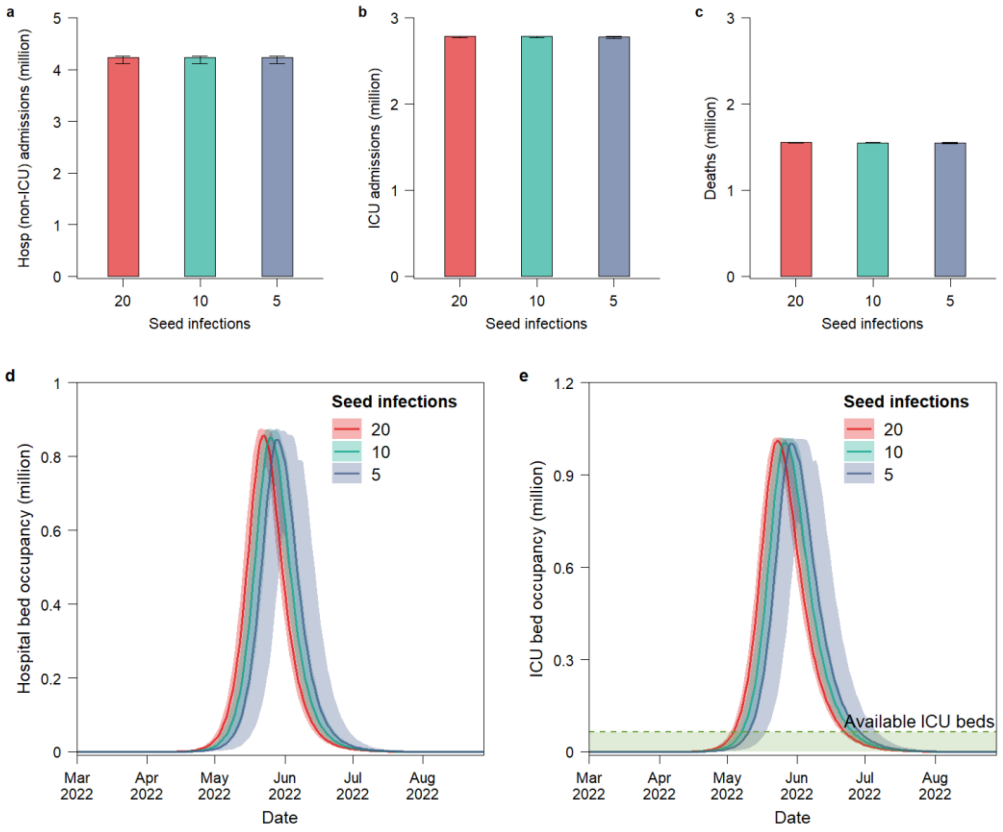

Supplementary Fig. 2 | Projected COVID-19 burden and demands in hospital and ICU beds in China under scenarios with less seeds of infections compared to baseline (20 seeds) scenario from March 2022 to September 2022. a, Cumulative hospital (non-ICU) admissions. **b**, Cumulative ICU admissions. **c**, Cumulative deaths. **d**, Daily demand in hospital (non-ICU) beds. **e**, Daily demand in ICU beds. In panel **e**, the green dashed line indicates the existing number of ICU beds in China. All data are presented as median with 2.5% and 97.5% quantiles of n=200 simulations.

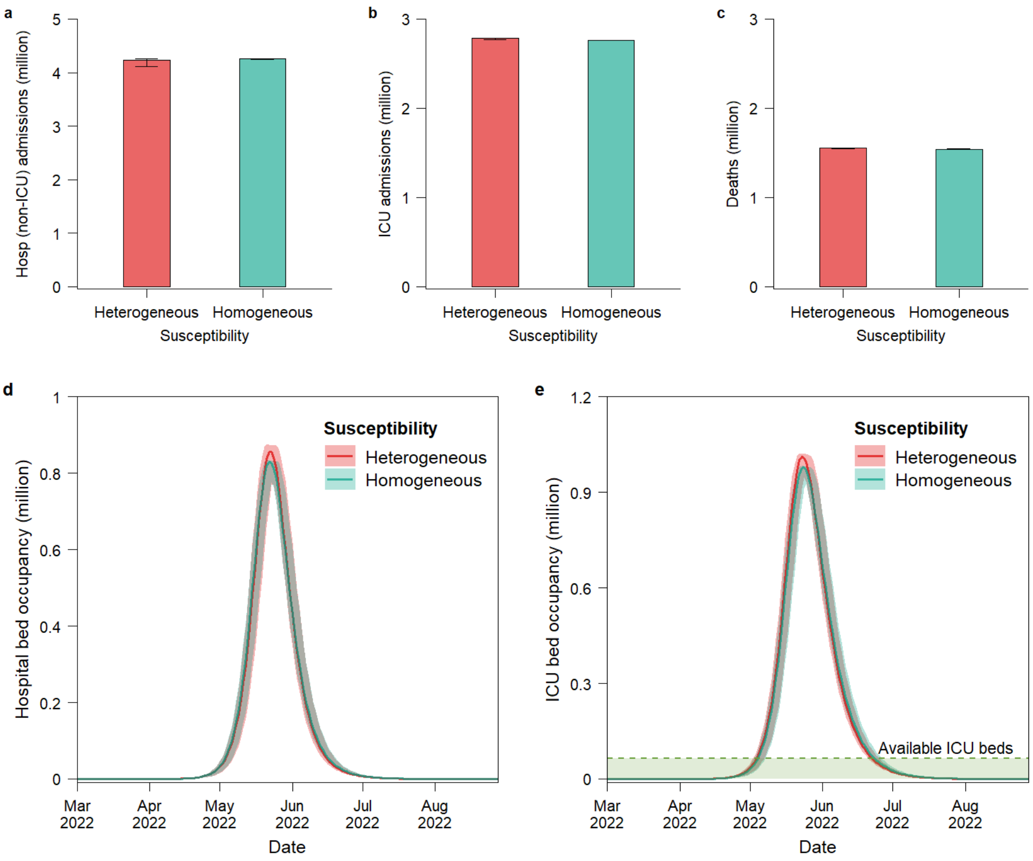

Supplementary Fig. 3 | Projected COVID-19 burden and demands in hospital and ICU beds in China under homogeneous susceptibility to infection across age group scenario compared to baseline (heterogeneous susceptibility) scenario from March 2022 to September 2022. a, Cumulative hospital (non-ICU) admissions. **b**, Cumulative ICU admissions. **c**, Cumulative deaths. **d**, Daily demand in hospital (non-ICU) beds. **e**, Daily demand in ICU beds. In panel **e**, the green dashed line indicates the existing number of ICU beds in China. All data are presented as median with 2.5% and 97.5% quantiles of *n*=200 simulations.

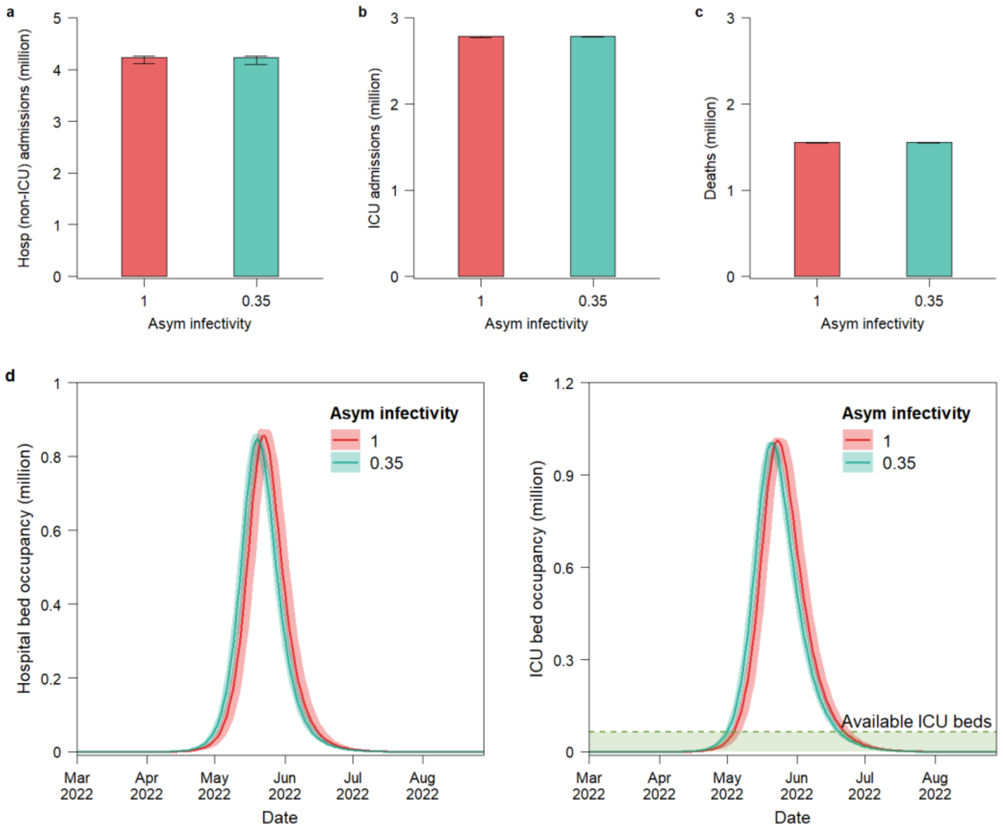

Supplementary Fig. 4 | Projected COVID-19 burden and demands in hospital and ICU beds in China under heterogeneous infectivity scenario compared to baseline (κ=1, equal infectivity) scenario from March 2022 to September 2022. a, Cumulative hospital (non-ICU) admissions. **b**, Cumulative ICU admissions. **c**, Cumulative deaths. **d**, Daily demand in hospital (non-ICU) beds. **e**, Daily demand in ICU beds. In panel **e**, the green dashed line indicates the existing number of ICU beds in China. Asym infectivity indicates parameter κ, where κ=0.35 denotes asymptomatic individuals are 65% less infectious than symptomatic individuals, and κ=1 denotes equal infectivity. All data are presented as median with 2.5% and 97.5% quantiles of n=200 simulations.

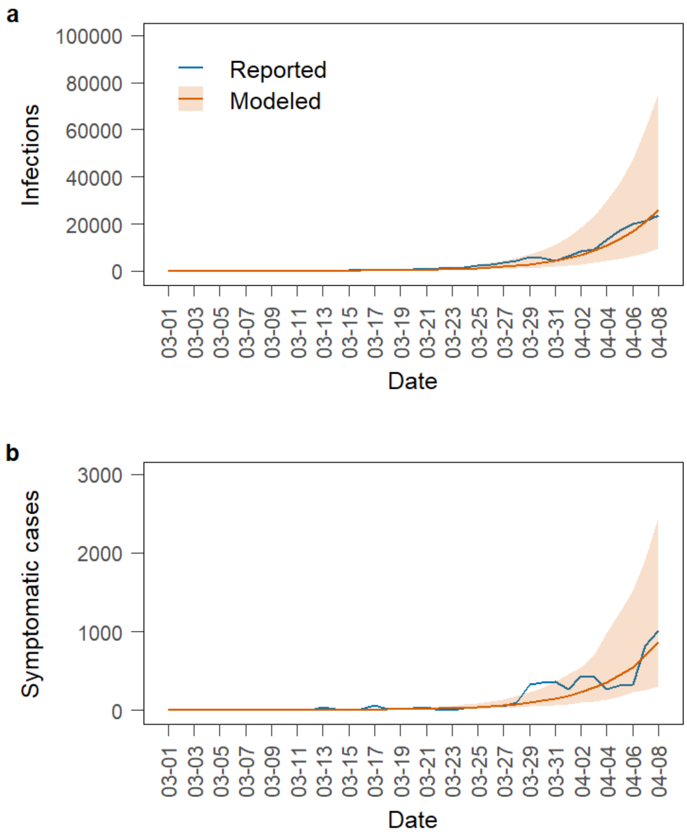

Supplementary Fig. 5 | Calibrating model outputs to the observed epidemic curves in Shanghai using a shorter generation time (T_g=4.7 days). a, Confirmed infections. **b**, Symptomatic cases. All data are presented as median with 2.5% and 97.5% quantiles of *n*=200 simulations.

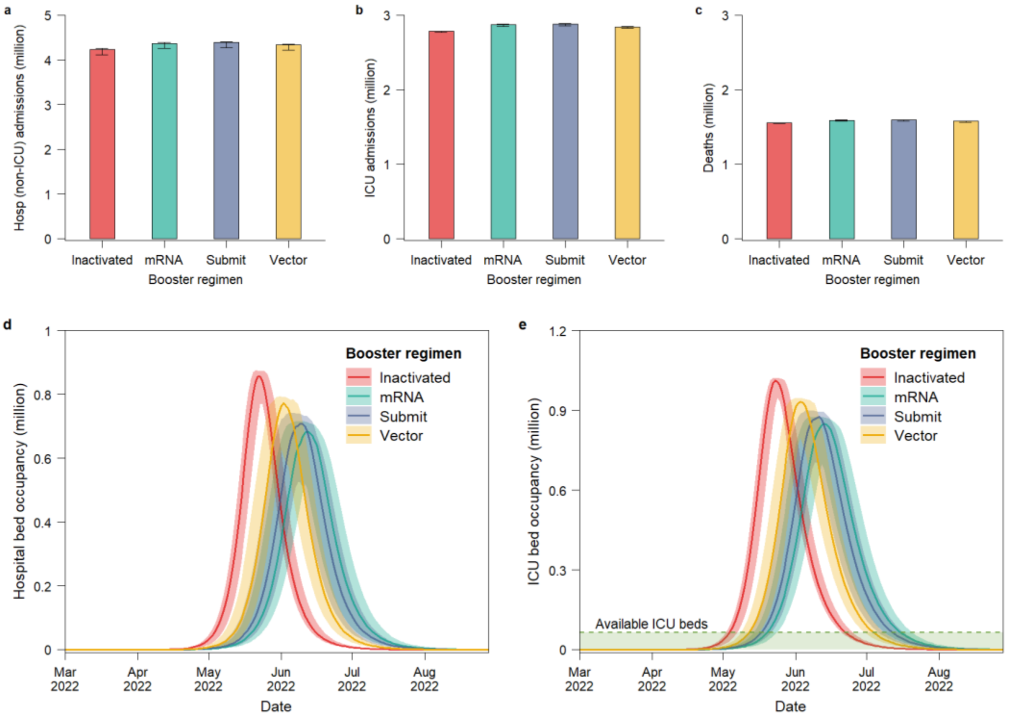

Supplementary Fig. 6 | Projected COVID-19 burden and demands in hospital and ICU beds in China for heterologous booster scenarios compared to baseline (homologous booster) scenario. The first row shows the total hospital admissions (**a**), ICU admissions (**b**), and deaths (**c**) over a 6-month simulation period. **d**, Daily demand in hospital beds for homologous booster vaccination using inactivated vaccines and heterologous booster vaccinations using mRNA, subunit, and vector vaccines scenarios. **e**, Daily demand in ICU beds for homologous booster vaccination using inactivated vaccines and heterologous booster vaccinations using mRNA, subunit, and vector vaccines scenarios. The green dashed line indicates the existing number of ICU beds in China. All data are presented as median with 2.5% and 97.5% quantiles of n=200 simulations.

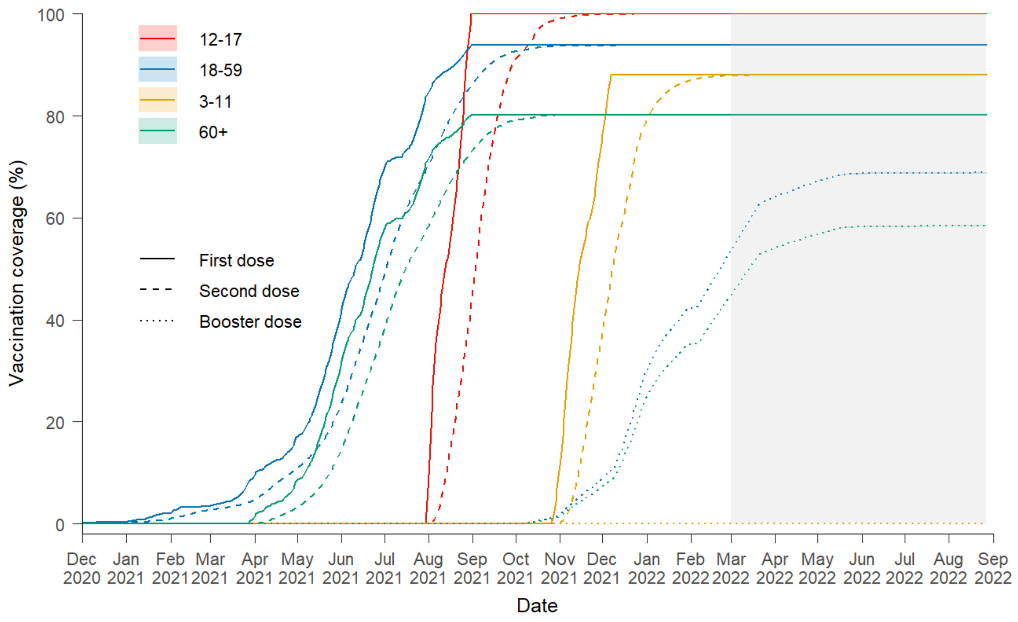

Supplementary Fig. 7 | Cumulative vaccine coverage by age in China shown for the first (solid lines), second (dashed lines), and booster (dotted lines) doses administered in the baseline scenario. The observed data until March 1, 2022 are presented, while the forward data are projected until August 31, 2022, in which period the booster vaccination rate is competing against the Omicron variant transmission.
